# Novel Linkages Between Bacterial Composition of Hindgut and Host Metabolic Responses to SARA Induced by High-Paddy Diet in Young Goats

**DOI:** 10.3389/fvets.2021.791482

**Published:** 2022-01-20

**Authors:** Kaijun Wang, Qiongxian Yan, Ao Ren, Mengli Zheng, Peihua Zhang, Zhiliang Tan, Chuanshe Zhou

**Affiliations:** ^1^CAS Key Laboratory of Agro-Ecological Processes in Subtropical Region, National Engineering Laboratory for Pollution Control and Waste Utilization in Livestock and Poultry Production, Hunan Provincial Key Laboratory of Animal Nutritional Physiology and Metabolic Process, Institute of Subtropical Agriculture, Chinese Academy of Sciences, Changsha, China; ^2^Hunan Provincial Key Laboratory for Genetic Improvement of Domestic Animal, College of Animal Science and Technology, Hunan Agricultural University, Changsha, China; ^3^University of Chinese Academy of Sciences, Beijing, China

**Keywords:** high paddy diet, SARA, bacterial diversity, goat, intestinal health

## Abstract

At present, feeding a high-corn diet to goats is used to provide enough protein and energy supply to meet their higher dietary requirements. In fact, because corn grain is commonly scarce in the traditional rice cropping region of southern Asia, paddy is thereby used as an alternative feed applied in goat diets. However, the effects of the high paddy proportion on the microbiota and metabolites of the intestine are unclear. Here, we investigate the effects of high paddy proportion on bacterial community, potential function, and metabolic reaction in the cecum of goats. Sixteen Liuyang black goats were divided into two groups fed either a normal-paddy (NP) diet (55% concentrate) or a high-paddy (HP) diet (90% concentrate) for 5 weeks. Total short-chain fatty acid (SCFA) concentration was higher in the hindgut chyme of the HP-fed goats than in that of the NP-fed goats (*p* = 0.001). The acetic proportion was significantly decreased and the propionic proportion was increased in the HP group (*p* < 0.05). The HP diet decreased the value of pH, lactic acid concentration, and lactate dehydrogenase activity but increased the activity of alanine aminotransferase, aspartate aminotransferase, alkaline phosphatase, and amylase, together with lipopolysaccharide concentration in the hindgut chyme of goats (*p* < 0.05). The abundance rates of the *Eubacterium_coprostanoligenes_group* was increased (*p* = 0.050), whereas the abundance of *Prevotellaceae_UCG_004, dgA-11_gut_group, Christensenellaceae_R-7_group, Ruminococcaceae_UCG-010*, and *Desulfovibrio* were significantly decreased with the HP diet (*p* < 0.05). These results suggested that the HP diet altered the microbiota and metabolites, which negatively modified intestinal epithelial health in goats.

## Introduction

Diet is a critical regulator that affects the composition of intestinal microbiota (1, 2), and a high-concentrate diet supplies adequate nutrients to meet the higher performance requirements for ruminants (3–5). Besides, rapid weight gain can be achieved by feeding goats a diet of 50–65% corn (6). However, in goats, a diet of highly fermentable carbohydrate increases metabolic diseases such as subacute ruminal acidosis (SARA) (7, 8). The presence of SARA is a major issue in terms of both productivity and animal welfare, and the gastrointestinal fermentation increased concentrations of short-chain fatty acid (SCFA) during SARA in dairy cows and goats (9, 10). Accumulation of SCFA production and digestion decreased the ruminal pH and led to SARA, which can decrease dry matter intake (DMI) and fiber absorption, increase the risk of poor health, and change milk nutrition and meat quality (11).

Increases of the dietary concentrate can alter the rumen and gut bacteria, but the effects of different dietary factors range diversely in dairy and beef cattle (12, 13). The intestinal microbes (such as Bacteroides, Firmicutes, and Proteobacteria) (14, 15) played a key role in regulating the immune system (16), some metabolic diseases (17), and inflammation (18). And a high-concentrate diet uses corn as the main concentrate (6, 19). In fact, since there is a shortfall of corn in the traditional paddy region of southern Asia, paddy is used as alternative feeds for goat models. Moreover, we did not know that the intestinal metabolism and microbial changes in goats due to a high-paddy (HP) diet induced SARA. The cecum is the second largest fermentation organ in ruminants after the rumen. Thus, we speculated that the higher SCFA in the cecum of the HP-fed goats, as compared to the normal-paddy (NP)-fed goats, may be due to the fact that a large number of carbohydrates were fermented in the cecum. These reports are essential to strengthen the understanding of the relationship between the HP diet and the cecal microbiota of goats, and this may lay the foundation for preventing the development of SARA.

In this study, 16 Liuyang goats were selected as the experimental model from the Liuyang animal cooperation base of the Institute of Subtropical Agriculture (ISA). These were divided into two groups fed an NP diet (55% concentrate) or an HP diet (90% concentrate) for 5 weeks. We hypothesized that the HP diet may cause variations in gut fermentation and microbiota activities and that these changes might cause cecal tissue damage in goats. Therefore, the purpose of this study was to investigate these changes in the cecal microbes, fermentation products, metabolites, and histomorphology during the feeding of an HP diet. In addition, we also assessed the relationship between changes in the cecal fermentation and microbial components and the metabolites of goats. The significance of this study is to reveal the underlying mechanism on exhaustive variation of the cecal bacteria caused by the HP diet.

## Experimental Design

The study was conducted according to the guidelines of the Animal Care Committee and approved by the ISA, Chinese Academy of Sciences, Changsha, China (ISA-201603).

### Animals, Diets, and Management

In this study, we randomly selected 16 healthy male goats (6 months old, 15.3 ± 1.7 kg), which were divided into two groups fed an NP diet (concentrate: forage = 55:45) or an HP diet (concentrate: forage = 90:10); the paddy and other concentrates were crushed together and stirred to mix well. The diet composition included paddy straw, the most commonly used forage in South China, selected as the roughage for goats, whereas paddy was the main concentrate, supplemented with soybean meal and wheat bran. Before the diets were formulated, the paddy straw was chopped to approximately 2 cm in length. The concentrate for this experiment was provided by the Hunan Lifeng Bio-Technology Company Ltd. (Changsha, China), and concentrate ingredients were consistent with a previous study (8). The proportion of rice straw in the NP and HP groups was 45:10. The concentrate of the experiment was composed of rice with shell, soybean meal, wheat bran, fat powder, calcium carbonate, calcium bicarbonate, and sodium chloride. The premix composition of the diet per kg was as follows: 68 mg FeSO_4_·H_2_O, 44 mg CuSO_4_·5H_2_O, 411 μg CoCl_2_·6H_2_O, 1.70 mg KIO_3_, 211 mg MnSO_4_·H_2_O, 126 mg ZnSO_4_·H_2_O, 56 μg Na_2_SeO_3_, 462 mg MgSO_4_·7H_2_O, 737 IU vitamin A, 8.29 mg vitamin E, 4.0 g NaHCO_3_, and 5.1 g carrier zeolite powder. The nutrient levels were measured values. The crude protein and crude fat were 17.6 and 6.01%, respectively, in the HP group, which were higher than those in the NP group. The crude ash, neutral detergent fiber, and acid detergent fiber in the HP group were lower than those in the NP group. The experiment lasted 35 days, and the goats were fed daily at 08:00 and 18:00 in a separate metabolic cage. All the goats had free access to water and feed intake of each goat was recorded daily. And the goats were housed in a well-ventilated room with controlled humidity and temperature.

### Sampling and Collection

At the end of the experiment on Day 35, six animals were eventually selected from each group and slaughtered at random. The cecal chyme was gathered and stored separately at −80 or −20°C for DNA extraction, and the metabolic indicators and SCFA were detected. Cecal segments were taken for observation of intestinal tissue morphology. Samples of the cecal segments from goats were fixed in formalin, and the tissues were dehydrated and embedded following standard procedures, and specimens in paraffin blocks were cut into 5 mm sections and stained with hematoxylin and eosin. Three slides per goat, two images per slide, and a total of 36 replicates per group were harvested. The representative photographs of the cecal morphology were collected using an optical microscope (Pannoramic DESK, P-MIDI, P250, 3DHISTECH, Hungary) with a Pannoramic scanner.

### Fermentation Parameters and Metabolites in the Cecal Chyme

The SCFA was analyzed from chromatograph peak areas using gas chromatography (Agilent 7890A, USA), according to the method described in our previous work (20). Meanwhile, the pH value of the cecal chyme fluid was determined using a pH meter (model PHS-3C, Shanghai Precision Science Instrument Co., Ltd., China). The cecal biochemical components including LACT, AST, LDH, ALT, AMY, and ALP were determined using an Automatic Biochemistry analyzer (Cobas c 311, Roche). Lipopolysaccharide (LPS) in the cecum was detected using a corresponding enzyme-linked immunosorbent assay (ELISA) kit (Jiangsu Yutong Biological Technology Co., Ltd. Yancheng, China).

### Genomic DNA Isolation and Polymerase Chain Reaction (PCR) Amplification

Genomic DNA isolation of the cecal contents was performed according to the instructions of a DNA Stool Mini Kit (Qiagen, Germany). The bacterial universal V3–V4 region of the 16S RNA gene was amplified according to PCR barcoded primers 338F (5′-ACTCCTACGGGAGGCAGCAG-3′) and 806R (5′-GGACTACHVGGG TWTCTAAT-3′). PCR was run in a 20 μl volume, containing 250 μM dNTP, 1 × FastPfu buffer, 0.1 μM of each primer, 1 U FastPfu polymerase (Beijing Trans Gen Biotech, China), and 10 ng template DNA. PCR was run at 95°C for 2 min, followed by 30 cycles of 95°C for 30 s, annealing at 55°C for 30 s, 72°C for 30 s, and a final extension at 72°C for 5 min.

### Illumina MiSeq Sequencing

PCR products were tested using 2% agarose gel electrophoresis and purified with AxyPrep DNA Purification Kit (Axygen Biosciences, Union City, USA). The PCR products were visualized on agarose gels and quantitatively determined using a QuantiFluor-ST fluorometer (Promega, USA) and Pico Green dsDNA Quantitation Reagent (Invitrogen, USA). The purified amplicon was subjected to equimolar pooling and paired-end sequenced on the Illumina MiSeq platform (Allwegene, Beijing, China) according to the standard procedure. The *16S rRNA* amplicon sequences have been deposited in the National Center for Biotechnology Information (NCBI) Sequence Read Archive (SRA) (http://www.ncbi.nlm.nih.gov/bioproject/738350) under accession number PRJNA738350.

### Bacterial Data Processing and Function Prediction

The sequencing data were analyzed. The raw FASTQ file was demultiplexed and quality-filtered using QIIME in the following order: (1) The reads (300 bp) were truncated at any site that obtained an average quality score of <20 over a 10-bp sliding window, and the truncated reads shorter than 50 bp were deleted; (2) exact barcodes were matched, and if two nucleotides mismatched in the primer matching, the reads containing ambiguous characters were discarded; and (3) the reads will be assembled if overlapping sequences were longer than 10 bp. The OTU was clustered using UPARSE according to 97% similarity, and chimeric sequences were identified and deleted using UCHIME. We used the Primer 6 software (UK) for hierarchical clustering analysis. The PCA was performed by Canoco 4.5. PICRUSt was used as a bioinformatics tool to predict the functional potentials of metagenomes using the 16S rRNA gene data (21). The OTU table was imported into PICRUSt for functional gene predication by referencing the KEGG database (Kyoto, Japan, http://www.genome.jp/kegg). The pathways included organismal systems, human diseases, and drug development, which were filtered out because these pathways did not reflect microbial functions.

### Statistical Analyses

The data were analyzed by SPSS 19.0 (USA, 2009). First, the data were evaluated through the Shapiro–Wilk test to check whether the variables exhibited a normal distribution. Then, the variables that showed a normal distribution and a non-normal distribution were analyzed by an independent-sample *t*-test and the Kruskal–Wallis test. Statistical significance was set at *p* < 0.05, and tendencies were set at 0.05 ≤ *p* ≤ 0.10.

## Results

### Morphology and Metabolic Parameters of the Hindgut

**Figure 1:**
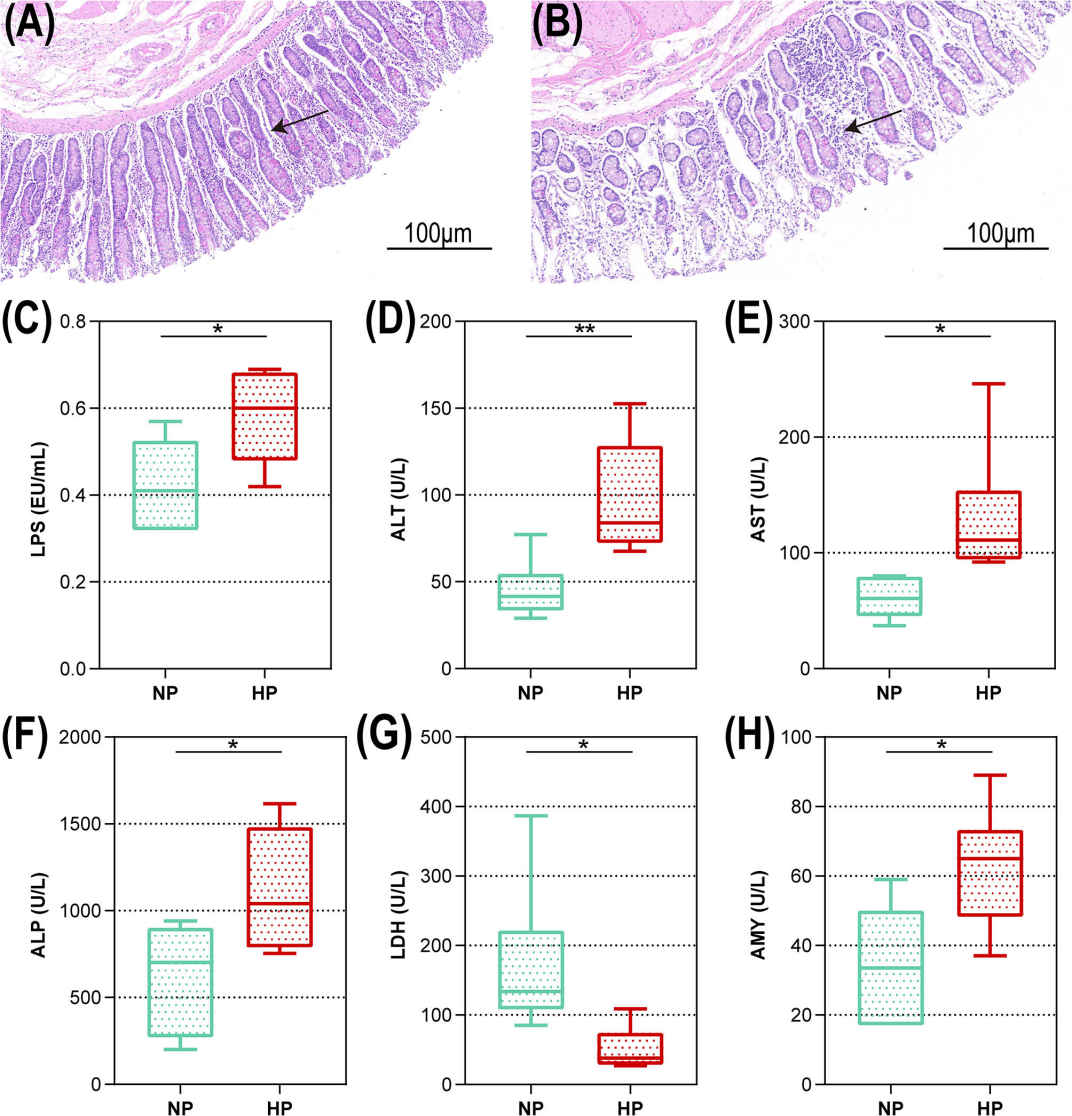
Intestinal morphology and cecal metabolites of goats fed NP and HP diet. Light microscopy cross-section of hindgut tissue from a representative NP-fed goat [**(A)** scale bar = 100 μm] or HP-fed goat [**(B)** scale bar = 100 μm]. **(C–H)** Metabolic parameters of the hindgut to goats fed by NP and HP diets. *0.01 < *p* < 0.05; **p < 0.01. The arrow indicate the observation of intestinal epithelial morphology.

The representative light micrograph of the intestinal morphology about the NP diet-fed and the HP diet-fed goats was shown in Figures 1A,B. The orifices of the crypts in the cecum were circular in outline, and the goat fed an NP diet had clear and well-organized microvillus clusters (Figure 1A). Conversely, the goats fed with an HP diet showed lower crypt density, sloughing of the epithelial surface, and irregular distribution compared with the goats fed an NP diet (Figure 1B). The cecal crypt depth of the HP diet-fed goats was significantly lower than that of the NP diet-fed goats (*p* < 0.05). As presented in Figure 1C, compared to the NP group, the HP group had increased LPS concentration in the cecum (*p* = 0.027). As shown in Figures 1D–H, only the concentration of LDH activity decreased significantly when the dietary concentrate ratio increased from 55 to 90% (*p* < 0.05). Compared with the NP diet-fed goats, the HP diet-fed goats had higher ALT, AST, ALP, and AMY activities in the cecal chyme (*p* < 0.05).

### Fermentation Parameters of the Hindgut

**Figure 2:**
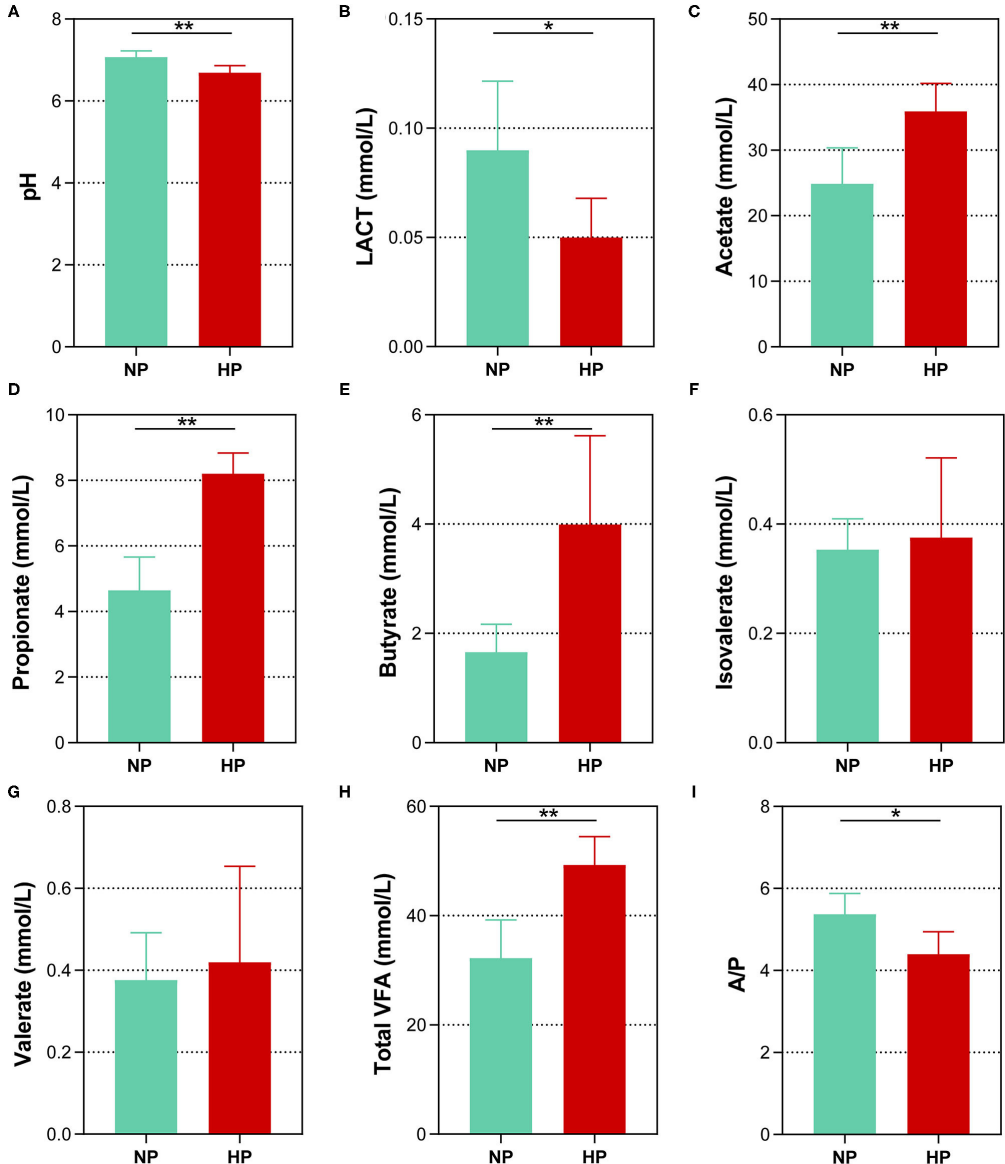
Effects of normal and high paddy diets on hindgut **(A)** pH, **(B)** LACT and **(C–H)** SCFA concentrations in goats. **(I)** A/P, Acetate: Propionate. *0.01 < *p* < 0.05; **p < 0.01.

Data of fermentation parameters in the cecum are displayed in Figure 2. The pH value in the cecal chyme declined dramatically when the dietary concentrate ratio increased from 55 to 90% (*p* < 0.01). The concentration of LACT activity notably declined when the dietary concentrate ratio increased from 55 to 90% (*p* < 0.05). In the cecum, the goats fed the HP diet had a significantly lower acetate-to-propionate ratio (A/P), when compared to the goats on the NP diet (*p* = 0.010). However, the HP dietary treatment significantly increased the concentrations of the TVFA, acetate, propionate, and butyrate (*p* < 0.01). In addition, no difference in the concentrations of isovalerate and valerate was found between the two dietary groups in the cecum (*p* > 0.05).

### Microbial Composition and Diversity

**Figure 3:**
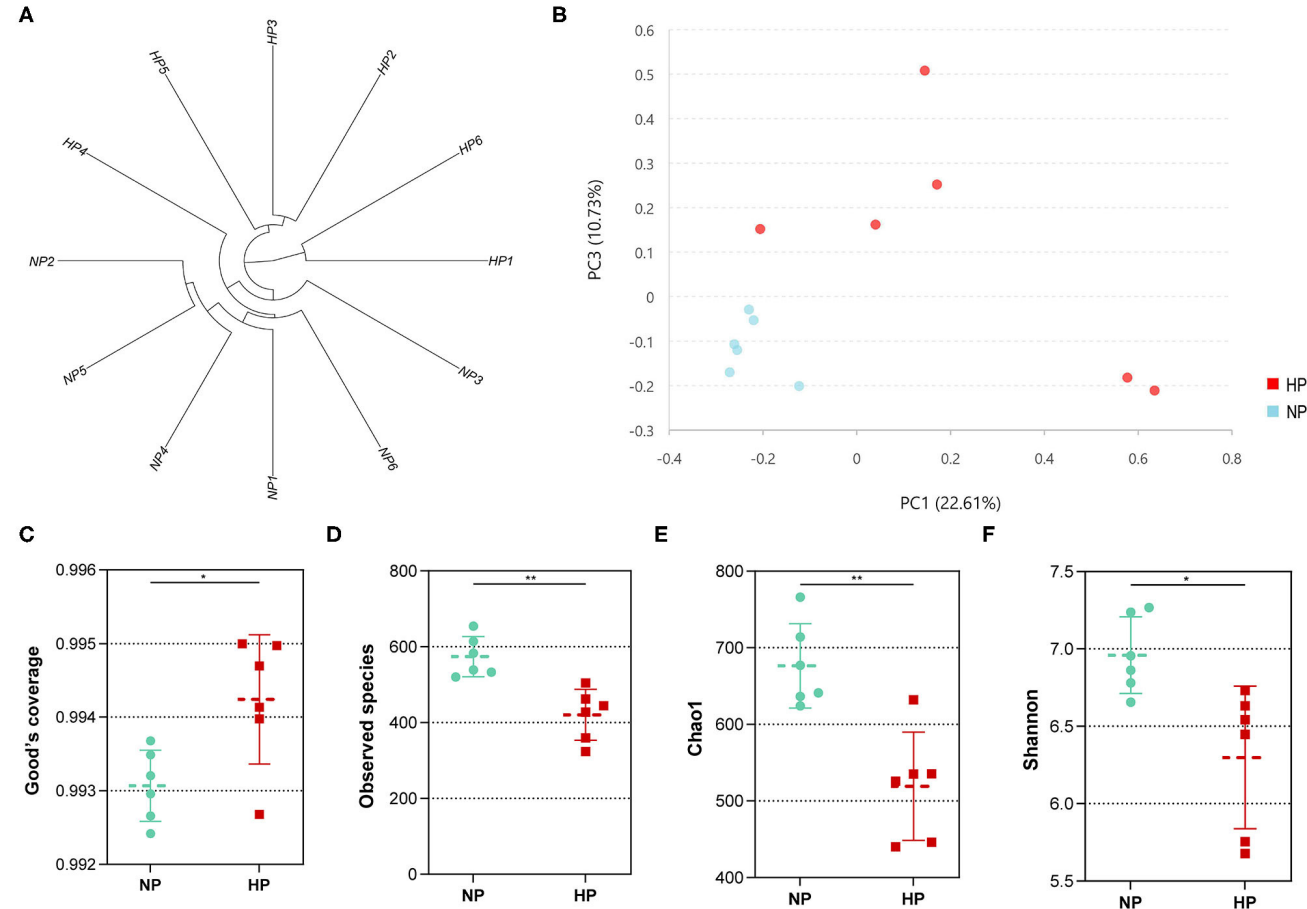
**(A)** Hierarchical clustering analysis of hindgut bacterial community (NP1–NP6 and HP1–HP6 are cecal samples of goats fed with NP and HP diets, separately). **(B)** PCA of cecal digestal microbiota. **(C–F)** Alpha diversity of microbiota of goats fed with NP and HP diets. *0.01 < *p* < 0.05; **p < 0.01.

In total, the cecum obtained 431,908 sequences after size filtering and chimera removal; each cecal sample had an average of 35,992 ± 5,622 sequences. Of the OTU numbers classified at 97% similarity, 1,276 were detected in the cecal samples, 1,133 in the NP group, 960 in the HP group, and 817 in both groups. The OTU by hierarchical clustering analysis presented that the samples in the NP group were separated from of those in the HP group (Figure 3A). In addition, similar results could be observed in the principal component analysis (PCA) (Figure 3B). When the ratio of concentrates increased from 55 to 90%, the Good coverage increased and Observed species of bacterial community decreased (*p* < 0.05) by the HP diet in the cecum (Figures 3C,D). Meanwhile, Chao 1 estimate and Shannon index of the bacterial community were also affected by the increase in dietary concentrate (Figures 3E,F).

### Bacterial Community Structure of the Hindgut

**Figure 4:**
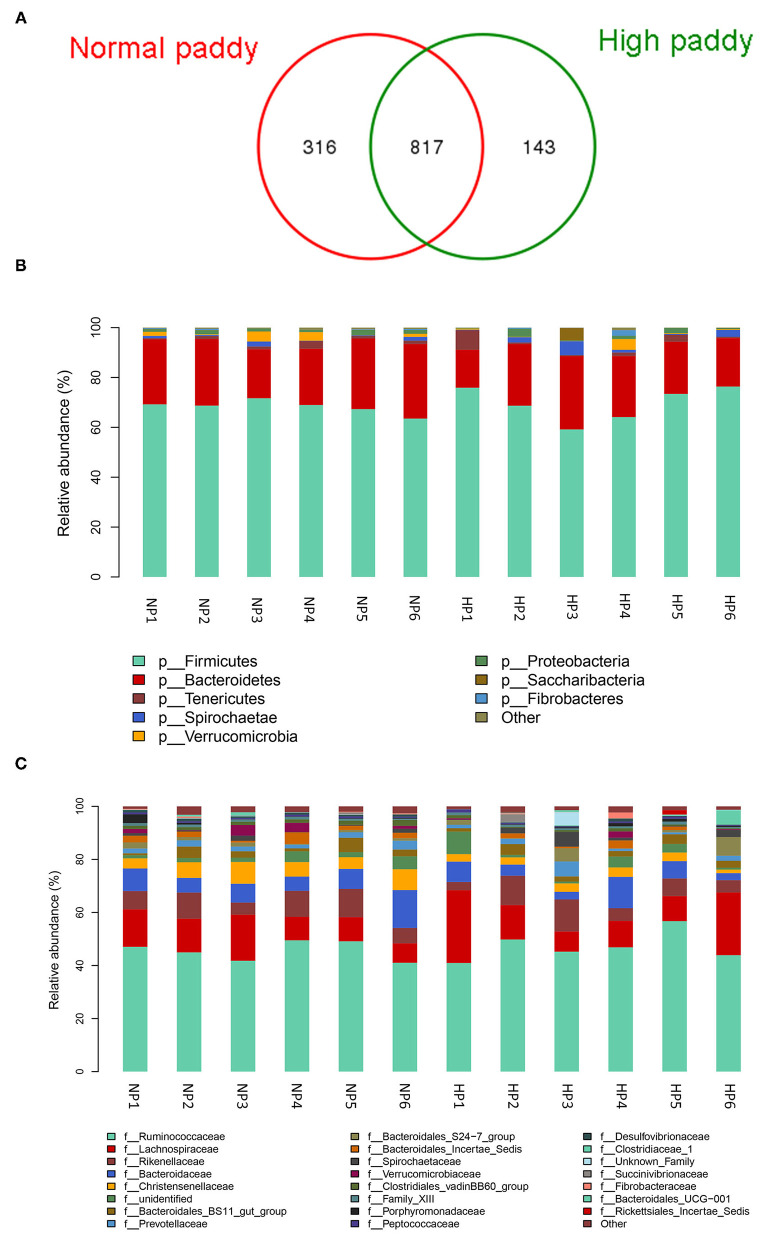
Effects of HP diet on hindgut microbiota of goats. **(A)** Venn diagram of the OTUs in different treatments. **(B)** Distribution of cecal microbiota at the phylum level in goats. **(C)** Distribution of cecal microbiota at the family level in goats (NP1–NP6 and HP1–HP6 are cecal samples of goats fed with NP and HP diets, separately).

The cecal microbial flora of two groups shared about 64% OTUs as shown in Figure 4A, and the NP group owned a larger number of unique OTUs. Firmicutes, Bacteroidetes, and Tenericutes were the main phyla in the cecum of goats, more than 90% of the total number of the cecal bacteria (Figure 4B). No difference was detected in the cecal bacteria of goats at the dominant phylum level (*p* > 0.05). The abundance of Lentisphaerae (*p* = 0.007) was lower in the HP diet-fed goats than in the NP diet-fed goats. At the family level in two groups, the cecal microbial flora was mainly composed of *Ruminococcaceae, Lachnospiraceae*, and *Rikenellaceae*, where *Bacteroidetes* consisted of *Bacteroidaceae, Bacteroidales_Incertae_Sedis, Prevotellaceae*, and *Rikenellaceae* and *Spirochaetaceae* consisted of *Spirochaetaceae* (Figure 4C). The proportion of *Christensenellaceae* (*p* = 0.003) and *Clostridiales_vadinBB60_group* (*p* = 0.011) dropped dramatically with the HP diet. No significant difference was found in the three primary families between the NP and HP groups (*p* > 0.05).

As shown in Table 1, down to the genus level, *Bacteroides, Christensenellaceae_R-7_group, Rikenellaceae_RC9_gut_group, Ruminococcaceae_UCG-010, Ruminococcaceae_UCG-005*, and *Eubacterium_coprostanoligenes_group* were primary genera in the NP and HP groups. The relative abundance of *Eubacterium_coprostanoligenes_group* was greater (*p* = 0.050) in the cecum of HP group when compared with the NP group. In contrast, the abundances of *Prevotellaceae_UCG-004, dgA-11_gut_group, Christensenellaceae_R-7_group, Ruminococcaceae_UCG-010*, and *Desulfovibrio* were less in the HP group than in the NP group (*p* < 0.05).

**Table 1:** Effects of NP and HP diets on the proportion of the cecal bacteria of goats at the genus level (%).

**Phylum**	**Genus**	**Abundance (%)**		***p*** **-value**
		**NP^a^**	**HP^b^**	
**Classification levels of bacteria**				
Bacteroidetes	*Bacteroides*	8.03 ± 3.28	5.95 ± 3.51	0.312
	*Phocaeicola*	2.21 ± 1.23	1.12 ± 1.18	0.148
	*Prevotella_1*	0.44 ± 0.68	0.86 ± 0.57	0.273
	*Prevotellaceae_UCG-003*	0.36 ± 0.48	0.88 ± 1.32	0.390
	*Prevotellaceae_UCG-004*	1.26 ± 1.01	0.02 ± 0.04	0.030
	*Alistipes*	2.13 ± 1.16	1.96 ± 2.08	0.862
	*dgA-11_gut_group*	0.69 ± 0.42	0.24 ± 0.23	0.045
	*Rikenellaceae_RC9_gut_group*	5.12 ± 2.19	4.84 ± 4.27	0.893
Firmicutes	*Christensenellaceae_R-7_group*	5.46 ± 1.65	2.65 ± 0.70	0.003
	*Clostridium_sensu_stricto_1*	0.04 ± 0.04	0.98 ± 2.16	0.334
	*Lachnospiraceae_AC2044_group*	1.77 ± 0.74	1.34 ± 1.20	0.469
	*Shuttleworthia*	0.09 ± 0.09	3.88 ± 6.16	0.192
	*Tyzzerella_4*	1.26 ± 0.51	0.68 ± 0.54	0.086
	*Eubacterium_coprostanoligenes_group*	4.93 ± 1.07	10.9 ± 5.77	0.050
	*Ruminococcaceae_NK4A214_group*	1.22 ± 0.41	1.45 ± 0.68	0.483
	*Ruminococcaceae_UCG-005*	16.7 ± 3.95	15.6 ± 4.61	0.669
	*Ruminococcaceae_UCG-010*	7.73 ± 1.50	4.67 ± 2.70	0.036
	*Ruminococcaceae_UCG-013*	2.61 ± 1.16	3.14 ± 1.60	0.526
	*Ruminococcaceae_UCG-014*	1.67 ± 0.79	3.08 ± 2.02	0.142
	*Saccharofermentans*	0.84 ± 1.53	0.22 ± 0.16	0.347
Proteobacteria	*Desulfovibrio*	0.76 ± 0.13	0.34 ± 0.39	0.031
Spirochaetae	*Treponema_2*	0.81 ± 0.73	1.93 ± 2.05	0.236
Verrucomicrobia	*Akkermansia*	1.72 ± 1.67	0.52 ± 0.93	0.156
	*Unidentified*	19.9 ± 2.72	20.1 ± 4.59	0.960

a *NP, normal-paddy diet*;

b *HP, high-paddy diet*.

### Function Prediction of Intestinal Microbiota

**Figure 5:**
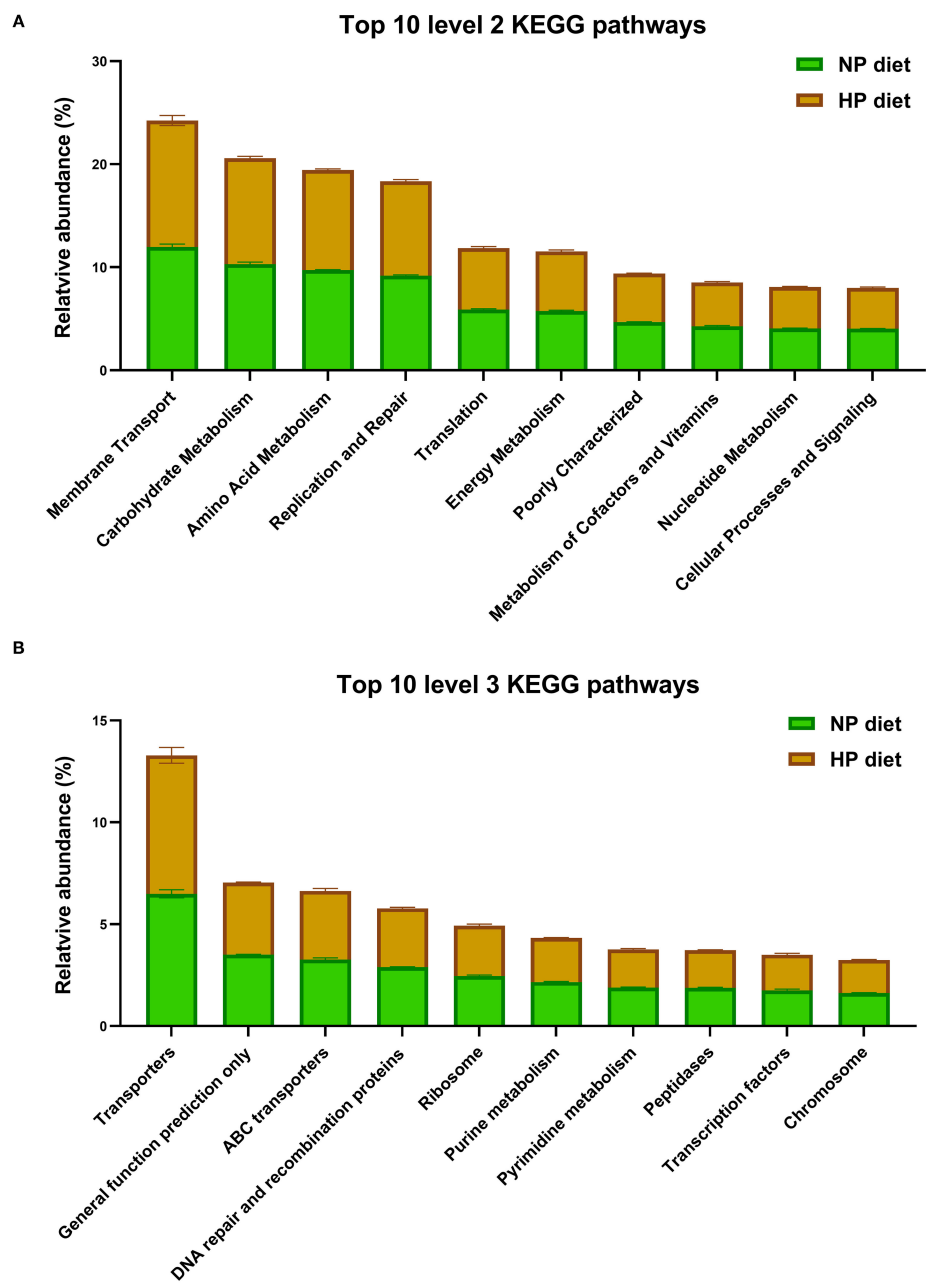
Top 10 predicted metagenomic functions at level 2 **(A)** and level 3 **(B)** of the KEGG pathways.

As shown in Figure 5A, the microbiota function was predicted by PICRUSt; the top 10 KEGG pathways included membrane transport; it was associated with environmental information processing. The other five pathways including metabolism of carbohydrates, amino acids, nucleotides, energy, cofactors, and vitamins were associated with metabolism. In addition, the pathways of translation, replication, and repair were associated with genetic information processing. The cellular processes and signaling were associated with signal transduction mechanisms at level 2.

In total, the PICRUSt predicted 152 individual pathways at level 3 in the cecum, and the primary top 10 pathways included three pathways about environmental information processing (as shown in Figure 5B), transporters, ATP-binding cassette (ABC) transporters, and transcription factors and four pathways about genetic information processes, including general function prediction only, DNA repair and recombination proteins, ribosomes, and chromosomes. Finally, three pathways of purine metabolism, pyrimidine metabolism, and peptidases were related to metabolism. As shown in Figure 5, KEGG level 2 and level 3 have no significant difference in the top 10 pathways (*p* > 0.05).

Data analysis showed that the HP diet affected the functional potentials in the cecal microbiota of growing goats (Table 2). Clearly, compared to the NP diet, the HP diet increased or tended to increase (*p* < 0.10) abundance of phenylalanine, tyrosine, and tryptophan biosynthesis (ko00400) and cysteine and methionine metabolism (ko00270) associated with amino acid metabolism. In contrast, the HP diet decreased butanoate metabolism (ko00650) associated with carbohydrate metabolism (*p* = 0.007) and tended to decrease (*p* < 0.10) abundances of histidine metabolism (ko00340) associated with amino acid metabolism and riboflavin metabolism (ko00740) associated with metabolism of cofactors and vitamins, respectively. There was no difference between pathways of biosynthesis of other secondary metabolites, cell motility, lipid metabolism, and many carbohydrate metabolism pathways among the NP and HP groups (*p* > 0.05).

**Table 2:** KEGG pathways that showed different abundances between the cecal digesta microbiota of goats fed with NP^a^ and HP^b^ diets.

**Level 2**	**Level 3**	**Pathway ID**	**NP**	**HP**	***p*** **-value**
Amino acid metabolism	Cysteine and methionine metabolism	ko00270	0.10 ± 0.007	0.98 ± 0.02	0.009
	Histidine metabolism	ko00340	0.63 ± 0.009	0.63 ± 0.01	0.093
	Phenylalanine, tyrosine, and tryptophan biosynthesis	ko00400	0.91 ± 0.01	0.93 ± 0.02	0.041
	Valine, leucine, and isoleucine biosynthesis	ko00290	0.80 ± 0.01	0.81 ± 0.009	0.461
	Valine, leucine, and isoleucine degradation	ko00280	0.19 ± 0.004	0.18 ± 0.02	0.861
Biosynthesis of other secondary metabolites	Novobiocin biosynthesis	ko00401	0.14 ± 0.004	0.14 ± 0.006	0.657
	Tropane, piperidine, and pyridine alkaloid biosynthesis	ko00960	0.12 ± 0.004	0.11 ± 0.008	0.367
Carbohydrate metabolism	Butanoate metabolism	ko00650	0.67 ± 0.01	0.63 ± 0.03	0.007
	Galactose metabolism	ko00052	0.70 ± 0.02	0.71 ± 0.04	0.794
	Pentose and glucuronate interconversions	ko00040	0.48 ± 0.01	0.49 ± 0.02	0.741
	Pentose phosphate pathway	ko00030	0.85 ± 0.04	0.87 ± 0.04	0.267
	Pyruvate metabolism	ko00620	1.09 ± 0.02	1.08 ± 0.02	0.377
	Starch and sucrose metabolism	ko00500	1.00 ± 0.02	1.04 ± 0.06	0.243
Cell motility	Bacterial chemotaxis	ko02030	0.63 ± 0.04	0.65 ± 0.05	0.473
	Flagellar assembly	ko02040	0.52 ± 0.04	0.54 ± 0.05	0.470
Glycan biosynthesis and metabolism	Other glycan degradation	ko00511	0.24 ± 0.02	0.25 ± 0.01	0.395
Lipid metabolism	Fatty acid biosynthesis	ko00061	0.50 ± 0.01	0.51 ± 0.02	0.875
	Glycerolipid metabolism	ko00561	0.38 ± 0.02	0.40 ± 0.02	0.358
Metabolism of cofactors and vitamins	Nicotinate and nicotinamide metabolism	ko00760	0.42 ± 0.01	0.43 ± 0.01	0.117
	Riboflavin metabolism	ko00740	0.23 ± 0.01	0.22 ± 0.01	0.092
	Vitamin B6 metabolism	ko00750	0.20 ± 0.01	0.19 ± 0.01	0.385
Signal transduction	Two-Component system	ko02020	1.56 ± 0.02	1.55 ± 0.07	0.633
Xenobiotics biodegradation and metabolism	Nitrotoluene degradation	ko00633	0.11 ± 0.01	0.10 ± 0.01	0.133
	Polycyclic aromatic hydrocarbon degradation	ko00624	0.09 ± 0.002	0.10 ± 0.01	0.783

a *NP, normal paddy diet*;

b *HP, high paddy diet*.

### Relationship Among the Intestinal Bacterial Community and Fermentation and Metabolic Parameters

**Figure 6:**
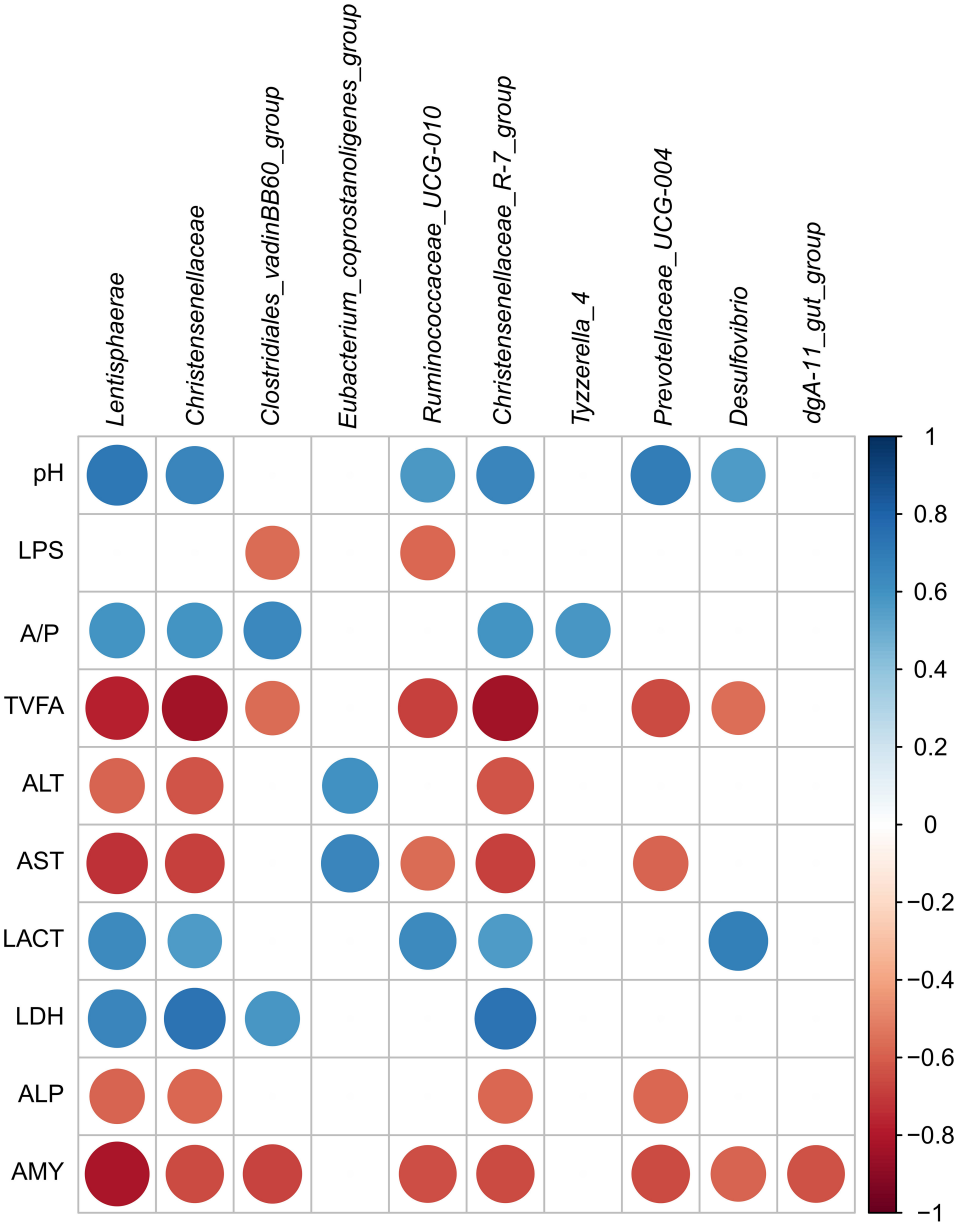
Correlation analysis among the intestinal pH, SCFA, and metabolites and associated microbiota composition of hindgut. Only results obtained for which the abundance was significantly associated with intestinal metabolites were shown. Cells were colored based on Pearson's correlation coefficient between the metabolites and associated microbiota composition in the hindgut (red and blue indicated negative and positive correlations, separately).

In the current study, the relationship of the microbiota, physiological parameters, and fermentation was analyzed by Pearson's correlation analysis. As presented in Figure 6, the pH was positively correlated with the genera *Lentisphaerae* (*r* = 0.713, *p* = 0.009), *Christensenellaceae_R-7_group* (*r* = 0.650, *p* = 0.022), and *Prevotellaceae_UCG-004* (*r* = 0.696, *p* = 0.012); however, no genus was significantly negatively correlated to the pH in the cecum. The abundances of *Lentisphaerae* (*r* = 0.657, *p* = 0.020), *Christensenellaceae* (*r* = 0.734, *p* = 0.007), *Clostridiales_vadinBB60_group* (*r* = 0.580, *p* = 0.048), and *Christensenellaceae_R-7_group* (*r* = 0.734, *p* = 0.007) were positively correlated with LDH, and no genus was significantly positively correlated with the ALP and AMY in the cecum (*r* < 0.55). The abundances of *Ruminococcaceae_UCG-010* and *Christensenellaceae*_*R-7*_*group* were positively correlated with LACT (*r* > 0.55, *p* < 0.05), and they negatively correlated with TVFA, AST, and AMY (*r* < −0.55, *p* < 0.05). Besides, the majority of the genera were negatively correlated with the activity of TVFA (*r* < −0.55) except *Eubacterium_coprostanoligenes_group, Tyzzerella_4*, and *dgA-11*_*gut*_*group* (*r* > −0.55). The abundances of three taxa [*Lentisphaerae* (*r* = −0.587, *p* = 0.044), *Christensenellaceae* (*r* = −0.629, *p* = 0.028), and *Christensenellaceae_R-7_group* (*r* = −0.629, *p* = 0.028)] were negatively correlated with the ALT, and only *Eubacterium_coprostanoligenes_group* (*r* = 0.601, *p* = 0.039) was positively correlated with ALT in the cecum. Meanwhile, only *Eubacterium_coprostanoligenes_group* (*r* = 0.650, *p* = 0.022) was positively correlated with AST, and other genera were negatively correlated with the AST.

## Discussion

The influence of a high-concentrate diet on SCFA in the rumen and hindgut of dairy cows and goats has been widely explored (8, 9). However, no information is available on changes of SCFA in the cecum with an HP diet. As has been mentioned in this study, the A/P declined in the cecum of goats by an HP diet compared to an NP diet, which proved that cecum fermentation was developing toward propionate production. Finally, the HP diet could reduce the pH of the cecum and lead to accumulated TVFA concentration in the hindgut. Several factors might account for the overfermentation and intestinal injury in the HP group. Firstly, the high abundance of carbohydrate in the cecum of the HP-fed goats might raise the osmotic pressure of chyme. There was no doubt that the high osmotic pressure was a threat to the intestinal barrier and might raise the permeability of the cecum (22). Secondly, the acidity of the intestinal lumen is one of the most important factors that determine the state of the gut barrier. Argenzio and Meuten revealed that a lower environmental pH value can stimulate the SCFA to enter non-glandular mucosal cells and acidify the cells, destroying the Na transport that depends on chloride and leading to cell necrosis (23). So, the study revealed the adverse effects of HP diet fed on the integrity of the cecal epithelium of goats and that the HP diet could raise the risk of poor health in goats.

Many studies reported that using a high-concentrate diet to feed ruminants would elevate free LPS concentration in the gastrointestinal tract, which could damage the epithelium of gastrointestinal tissue (24, 25). In this study, the LPS concentration in the cecal chyme was significantly negatively correlated with the proportion of Gram-negative bacteria, including the taxa *Prevotellaceae_UCG-004* (*r* = −0.131), *dgA-11_gut_group* (*r* = −0.356), and *Desulfovibrio* (*r* = −0.466). In addition, the three taxa were much higher in the NP group compared to the HP group. Our data proved that the HP diet in goats could cause the lysis and death of some Gram-negative bacteria in the hindgut, therefore raising the LPS concentration in the cecum. It also caused damage to the cecal epithelium. The enzyme levels of ALP, ALT, and AST increased with some damage in membrane permeability (26); this was also one of the reasons why our goats had damage to their cecal epithelium in the HP group. The *Christensenellaceae*_*R-7*_*group* and *Prevotellaceae*_*UCG-004* abundances were negatively correlated with the concentration of ALP; this indicated that the two genera might be involved in the ALP metabolism of the cecum of HP diet-fed goats. However, the role of these genera in the intestine and how they associated with the intestine needed to be clarified. The enzyme of LDH was released during intestinal tissue damage, and it was a marker of common injury and disease. Particularly, feedback inhibition of LDH could decrease the conversion of pyruvate to LACT at a high LACT level (27). Our research displayed that both LDH and LACT were decreased simultaneously by the HP diet. So, we speculated that LACT concentration and LDH activity were linearly proportional in the cecum. The HP diet increased the activity of amylase, and this was similar to the results that more starch intake increased the amylase activity in the pancreas and small intestine contents (28, 29). However, we found that *Christensenellaceae*_*R*-*7*_*group, Ruminococcaceae*_*UCG*-*010*, and *Prevotellaceae*_*UCG*-*004* were correlated negatively with the enzyme of AMY in the cecum. The results may indicate that the higher AMY activity may be partly related to the decrease of the three genera in the HP group.

Gut microbes play a special role in host nutrition metabolism and absorption, maintenance of the intestinal barrier structure, immune regulation, and resistance to pathogen (30). In accordance with the previous study that proved feeding a high-concentrate diet could decrease the microbiota diversity in the gastrointestinal tract of cattle (31), our research also proved a decline in the microflora of the cecum of goats fed an HP diet. A separation between colonic samples of the two groups could be best observed by hierarchical clustering analysis, and the results of the PCA revealed the difference in bacterial diversity composition between the NP and the HP groups. In addition, the results of the Chao 1 estimate and Shannon indices in the HP group were observed to be decreased; it further revealed the difference in bacterial diversity composition between the NP and HP groups, also indicating that an HP diet changed the bacterial diversity in the cecum. Changes in the content of the dietary nutrient between the NP and the HP groups might have resulted in the variations in colonic microbial response. The findings of Wetzels might explain this situation (32); the low cecal pH value might reduce some cecal chyme bacterial richness due to their sensitivity to low pH in this study, leading to the proliferation of some low-pH-tolerant bacteria in the cecum. Hence, feeding goats with the HP diet tended to decrease the bacterial diversity in the cecum, and the HP diet caused a negative effect on the healthy hindgut ecosystem. Compared to previous studies that reported on the intestinal bacterial communities of ruminants (7, 33), our results revealed that Firmicutes were the most dominant phylum, with 69.34% of total sequences, from the hindgut chyme of goats. The Firmicutes are well-known for fermentative metabolism and protein and amino acid degradation (34). So, the massive Firmicutes in the cecal chyme microbiota makes it known that Firmicutes have a key role in the utilization of nutrients such as protein and amino acids. Consistent with an earlier study (35), we confirmed that the phylum Bacteroidetes of Gram-negative bacteria was the second most abundant bacterial community. A study of Khafipour and Li concluded that a majority of LPS produced in the chyme was derived from *Bacteroides* spp. (36). In the present study, there was no difference in *Bacteroides* level difference between goats in the two groups, so this phenomenon could not explain that the increased LPS level in the cecum was due to the HP diet. Jenkins et al. (37) found that the *Christensenellaceae* family played a key role in maintaining gut structure and function. We detected that a decreased cecal bacterial response to *Christensenellaceae_R-7_group* significantly was caused by feeding an HP diet to goats. Our results somehow explained why the epithelial structure of the cecum tissue was damaged in the HP group. *Ruminococcus* was the primary genus in the hindgut of sheep (38), and it could degrade starch, xylan, and other complex polysaccharides (39). For the study, we found a majority of *Ruminococcaceae* in the HP group. It was possible that the HP diet provided suitable nutrient conditions for the growth of *Ruminococcaceae*. Liu found that there were higher *Prevotella* and *Turicibacter* levels in the hindgut when goats were fed a high-concentrate (19). There were some varieties on the abundance of *Prevotellaceae*; the present study revealed that there was no difference on *Prevotella_1* and *Prevotellaceae_UCG-003* between the two groups, but *Prevotellaceae_UCG-004* showed a marked drop in the cecum due to the HP diet. The *Turicibacter* in the intestine can induce subclinical infection or other deleterious effects in the digestive tract (40); nevertheless, the *Turicibacter* population was <1% in the cecal chyme of goats in the current study. Previous research studies indicated that some *Clostridium* spp. were pathogenic factors of intestinal disease and could alter the intestinal barrier in animals (41, 42). The current study provided a detailed picture of the cecal chyme-associated bacterial community when feeding an HP diet and that it could inhibit the growth of intestinal bacterial communities.

The PICRUSt results showed that cecal microorganisms had various functions; the primary gene categories in the cecum-associated bacteria of goat were related to the pathways of carbohydrate and energy metabolism, replication and repair, and membrane transport; they were similar to the results of a previous study conducted in the colon of goats (7). The cecal bacteria had been predicted to have greater replication and repair capacity, which might cause the rapid turnover rate of microbe in the hindgut. The membrane transport system existed in all living organisms, and it was necessary to communicate with tissue, such as importing molecules into cells and discharging waste products from cells (43). Both diet and symbiotic bacteria determined the number of vitamins in the mammalian gut (44), so vitamins could not be synthesized by goats but could be synthesized by commensal bacteria or from the diet. In the pathway of energy metabolism, riboflavin and its active form act as cofactors in various enzymatic reactions, such as the TCA cycle and FAO (45). Sakurai had shown that riboflavin deficiency could decrease the acyl-CoA dehydrogenases activity (46), which participated in the dehydrogenation step of FAO, and riboflavin supplementation rescued the activity of these enzymes. The decrease of LDH activity might be related to the signaling process involved in the reduction of riboflavin metabolism in the HP group. Therefore, microbial functional potential predication identified that the HP diet influenced several pathways and that nutrient effectiveness affects the cecal metabolism, as well as bacterial structure and potential functions.

## Conclusion

In summary, the HP diet could regulate the growth of hindgut microflora, which may promote the risk of poor health in goats. This understanding is essential to elicit predictable changes in the gut microflora through nutritional strategies (such as dietary interventions) to promote the productivity and welfare of goats.

## Data Availability Statement

The datasets presented in this study can be found in online repositories. The names of the repository/repositories and accession number(s) can be found in the article/supplementary material.

## Ethics Statement

The animal study was reviewed and approved by the study was conducted according to Animal Care Committee and proved by the Institute of Subtropical Agriculture, Chinese Academy of Sciences, Changsha, China (ISA-201603).

## Author Contributions

ZT and CZ designed the experiment. QY and KW conducted the experiment. KW, AR, and MZ collected and analyzed data. KW wrote the manuscript. PZ and CZ revised the manuscript. All authors contributed to the article and approved the submitted version.

## Funding

This work was supported by the National Natural Science Foundation of China (Grant No. 31772632 and 31372342); Youth Innovation Team Project of ISA, CAS (2017QNCXTD_ZCS), China; and Hunan Provincial Science & Technology Department (2017JJ1028).

## Conflict of Interest

The authors declare that the research was conducted in the absence of any commercial or financial relationships that could be construed as a potential conflict of interest.

## Publisher's Note

All claims expressed in this article are solely those of the authors and do not necessarily represent those of their affiliated organizations, or those of the publisher, the editors and the reviewers. Any product that may be evaluated in this article, or claim that may be made by its manufacturer, is not guaranteed or endorsed by the publisher.

## References

[1] Ng SH Stat M Bunce M Simmons LW. The influence of diet and environment on the gut microbial community of field crickets. Ecol Evol. (2018) 8:4704–20. 10.1002/ece3.3977 29760910 PMC5938447

[2] Clements SJ Carding SR. Diet, the intestinal microbiota, and immune health in aging. Crit Rev Food Sci. (2018) 58:651–61. 10.1080/10408398.2016.1211086 27712080

[3] Boerman JP Potts S VandeHaar MJ Allen MS Lock AL. Milk production responses to a change in dietary starch concentration vary by production level in dairy cattle. J Dairy Sci. (2015) 98:4698–706. 10.3168/jds.2014-8999 25981075

[4] Pourazad P Khiaosa-Ard R Qumar M Wetzels SU Klevenhusen F Metzler-Zebeli BU. Transient feeding of a concentrate-rich diet increases the severity of subacute ruminal acidosis in dairy cattle. J Anim Sci. (2016) 94:726–38. 10.2527/jas.2015-9605 27065143

[5] Mao SY Huo WJ Zhu WY. Microbiome-metabolome analysis reveals unhealthy alterations in the composition and metabolism of ruminal microbiota with increasing dietary grain in a goat model. Environ Microbiol. (2016) 18:525–41. 10.1111/1462-2920.12724 25471302

[6] Tao S Duanmu Y Dong H Tian J Ni Y Zhao R. A high-concentrate diet induced colonic epithelial barrier disruption is associated with the activating of cell apoptosis in lactating goats. BMC Vet Res. (2014) 10:235. 10.1186/s12917-014-0235-2 25256013 PMC4180839

[7] Ye HM Liu JH Feng PF Zhu WY Mao SY. Grain-rich diets altered the colonic fermentation and mucosa-associated bacterial communities and induced mucosal injuries in goats. Sci Rep. (2016) 6:20329. 10.1038/srep20329 26841945 PMC4740883

[8] Wang KJ Zheng ML Ren A Zhou CS Yan QX Tan ZL. Effects of high rice diet on growth performance, nutrients apparent digestibility, nitrogen metabolism, blood parameters and rumen fermentation in growing goats. Kafkas Univ Vet Fak Derg. (2019) 25:749–55. 10.9775/kvfd.2019.21721

[9] Li S Khafipour E Krause DO Kroeker A Rodriguez-Lecompte JC Gozho GN. Effects of subacute ruminal acidosis challenges on fermentation and endotoxins in the rumen and hindgut of dairy cows. J Dairy Sci. (2012) 95:294–303. 10.3168/jds.2011-4447 22192209

[10] Metzler-Zebeli BU Schmitz-Esser S Klevenhusen F Podstatzky-Lichtenstein L Wagner M Zebeli Q. Grain-rich diets differently alter ruminal and colonic abundance of microbial populations and lipopolysaccharide in goats. Anaerobe. (2013) 20:65–73. 10.1016/j.anaerobe.2013.02.005 23474085

[11] Plaizier JC Krause DO Gozho GN McBride BW. Subacute ruminal acidosis in dairy cows: the physiological causes, incidence and consequences. Vet J. (2008) 176:21–31. 10.1016/j.tvjl.2007.12.016 18329918

[12] Mao S Zhang R Wang D Zhu W. Impact of subacute ruminal acidosis (SARA) adaptation on rumen microbiota in dairy cattle using pyrosequencing. Anaerobe. (2013) 24:12–9. 10.1016/j.anaerobe.2013.08.003 23994204

[13] Petri RM Schwaiger T Penner GB Beauchemin KA Forster RJ McKinnon JJ. Changes in the rumen epimural bacterial diversity of beef cattle as affected by diet and induced ruminal acidosis. Appl Environ Microbiol. (2013) 79:3744–55. 10.1128/AEM.03983-12 23584771 PMC3675914

[14] Vitetta L Briskey D Alford H Hall S Coulson S. Probiotics prebiotics and the gastrointestinal tract in health and disease. Inflammopharmacology. (2014) 22:135–54. 10.1007/s10787-014-0201-4 24633989

[15] Tojo R Suárez A Clemente MG de los Reyes-Gavilán CG Margolles A Gueimonde M. Intestinal microbiota in health and disease: role of bifidobacteria in gut homeostasis. World J Gastroenterol. (2014) 20:15163–76. 10.3748/wjg.v20.i41.15163 25386066 PMC4223251

[16] Zhang H Sparks JB Karyala SV Settlage R Luo XM. Host adaptive immunity alters gut microbiota. ISME J. (2015) 9:770–81. 10.1038/ismej.2014.165 25216087 PMC4331585

[17] Rothschild D Weissbrod O Barkan E. Environment dominates over host genetics in shaping human gut microbiota. Nature. (2018) 555:210–5. 10.1038/nature25973 29489753

[18] Koji A Wataru S Chengwei L Takaaki K Iori M Seiko N. Ectopic colonization of oral bacteria in the intestine drives T(H)1 cell induction and inflammation. Science. (2017) 358:359–65. 10.1126/science.aan4526 29051379 PMC5682622

[19] Liu H Xu TT Zhu WY Mao SY. High-grain feeding alters caecal bacterial microbiota composition and fermentation and results in caecal mucosal injury in goats. Br J Nutr. (2014) 112:416–27. 10.1017/S0007114514000993 24846282

[20] Jiao JZ Wu J Zhou CS Tang SX Wang M Tan ZL. Composition of ileal bacterial community in grazing goats varies across non-rumination, transition and rumination stages of life. Front Microbiol. (2016) 7:1364. 10.3389/fmicb.2016.01364 27656165 PMC5011132

[21] Langille MG Zaneveld J Caporaso JG McDonald D Knights D Reyes JA. Predictive functional profiling of microbial communities using 16S rRNA marker gene sequences. Nat Biotechnol. (2013) 31:814–21. 10.1038/nbt.2676 23975157 PMC3819121

[22] Gressley TF Hall MB Armentano LE. Ruminant nutrition symposium: productivity, digestion, and health responses to hindgut acidosis in ruminants. J Anim Sci. (2011) 89:1120–30. 10.2527/jas.2010-3460 21415422

[23] Argenzio RA Meuten DJ. Short-chain fatty acids induce reversible injury of porcine colon. Dig Dis Sci. (1991) 36:1459–68. 10.1007/BF01296816 1914771

[24] Liu JH Xu TT Liu YJ Zhu WY Mao SY. A high-grain diet causes massive disruption of ruminal epithelial tight junctions in goats. Am J Physiol Regul Integr Comp Physiol. (2013) 305:R232–41. 10.1152/ajpregu.00068.2013 23739344

[25] Emmanuel DG Madsen KL Churchill TA. Acidosis and lipopolysaccharide from *Escherichia coli* B: 055 cause hyperpermeability of rumen and colon tissues. J Dairy Sci. (2007) 90:5552–7. 10.3168/jds.2007-0257 18024746

[26] Rashid S Irshadullah M. Evaluation of antioxidant and oxidant status of goats (Capra aegagrus hircus) naturally infected with *Haemonchus contortus*. J Helminthol. (2019) 94:e36. 10.1017/S0022149X19000117 30761971

[27] Selwood T Jaffe EK. Dynamic dissociating homo-oligomers and the control of protein function. Arch Biochem Biophys. (2012) 519:131–143. 10.1016/j.abb.2011.11.020 22182754 PMC3298769

[28] Wang XB Ogawa T Suda S Kohzo Taniguchi S. Effects of nutritional level on digestive enzyme activities in the pancreas and small intestine of calves slaughtered at same body weight. Asian-Australas J Anim Sci. (1998) 11:375–80. 10.5713/ajas.1998.375

[29] Owsley WF Orr DE Tribble LF. Effects of age and diet on the development of the pancreas and the synthesis and secretion of pancreatic enzymes in the young pig. J Anim Sci. (1986) 63:497–504. 10.2527/jas1986.632497x 2428799

[30] Song PX Zhang RJ Wang XX He P Tan L Ma X. Dietary grape-seed procyanidins decreased post-weaning diarrhea by modulating intestinal permeability and suppressing oxidative stress in rats. J Agric Food Chem. (2011) 59:6227–32. 10.1021/jf200120y 21534629

[31] Khafipour E Li S Tun H Derakhshani H Moossavi S. Effects of grain feeding on microbiota in the digestive tract of cattle. Anim Front. (2016) 6:13–9. 10.2527/af.2016-001832271920

[32] Wetzels SU Mann E Metzler-Zebeli BU Wagner M Klevenhusen F Zebeli Q. Pyrosequencing reveals shifts in the bacterial epimural community relative to dietary concentrate amount in goats. J Dairy Sci. (2015) 98:5572–87. 10.3168/jds.2014-9166 26051320

[33] Plaizier JC Li S Tun HM Khafipour E. Nutritional models of experimentally-induced subacute ruminal acidosis (SARA) differ in their impact on rumen and hindgut bacterial communities in dairy cows. Front Microbiol. (2016) 25:2128. 10.3389/fmicb.2016.02128 28179895 PMC5265141

[34] Tang YQ Shigematsu T Morimura S Kida K. Microbial community analysis of mesophilic anaerobic protein degradation process using bovine serum albumin (BSA)-fed continuous cultivation. J Biosci Bioeng. (2005) 99:150–64. 10.1263/jbb.99.150 16233772

[35] Tao SY Tian P Luo YW Tian J Hua CF Geng YL. Microbiome-metabolome responses to a high-grain diet associated with the hind-gut health of goats. Front Microbiol. (2017) 8:1764. 10.3389/fmicb.2017.01764 28959247 PMC5603706

[36] Khafipour E Li S Plaizier JC Krause DO. Rumen microbiome composition determined using two nutritional models of subacute ruminal acidosis. Appl Environ Microbiol. (2009) 75:7115–24. 10.1128/AEM.00739-09 19783747 PMC2786511

[37] Jenkins S Waite I Mansfield J Kim J Pluske J. Relationships between diets different in fibre type and content with growth, *Escherichia coli* shedding, and faecal microbial diversity after weaning. Anim Prod Sci. (2015) 55:1451. 10.1071/ANv55n12Ab12528948418

[38] Wang J Fan H Han Y Zhao J Zhou Z. Characterization of the microbial communities along the gastrointestinal tract of sheep by 454 pyroseqencing analysis. Asian-Australas J Anim Sci. (2016) 1:100–10. 10.5713/ajas.16.0166 27383798 PMC5205584

[39] Chassard C Delmas E Robert C Lawson PA Bernalier-Donadille A. *Ruminococcus champanellensis sp*. nov., a cellulose-degrading bacterium fromhuman gut microbiota. Int J Syst Evol Microbiol. (2012) 62:138–43. 10.1099/ijs.0.027375-0 21357460

[40] Bosshard PP Zbinden R Altwegg M. Turicibacter sanguinis gen nov., sp. nov., a novel anaerobic, *Grampositive bacterium*. Int J Syst Evol Microbiol. (2002) 52:1263–6. 10.1099/00207713-52-4-1263 12148638

[41] Garcia JP Adams V Beingesser J Hughes ML Poon R Lyras D. Epsilon toxin is essential for the virulence of *Clostridium perfringens* type D infection in sheep, goats, and mice. Infect Immun. (2013) 81:2405–14. 10.1128/IAI.00238-13 23630957 PMC3697609

[42] Kellermayer R Dowd SE Harris RA Balasa A Schaible TD Wolcott RD. Colonic mucosal DNA methylation, immune response, and microbiome patterns in toll-like receptor 2-knockout mice. FASEB J. (2011) 25:1449–60. 10.1096/fj.10-172205 21228220 PMC3079304

[43] Konishi H Fujiya M Kohgo Y. Host–microbe interactions via membrane transport systems. Environ Microbiol. (2015) 17:931–7. 10.1111/1462-2920.12632 25286963

[44] Shibata N Kunisawa J Kiyono H. Dietary and microbial metabolites in the regulation of host immunity. Front Microbiol. (2017) 8:2171. 10.3389/fmicb.2017.02171 29163449 PMC5681998

[45] Huskisson E Maggini S Ruf M. The role of vitamins and minerals in energy metabolism and well-being. J Int Med Res. (2007) 35:277–89. 10.1177/147323000703500301 17593855

[46] Sakurai T Miyazawa S Furuta S Hashimoto T. Riboflavin deficiency and beta-oxidation systems in rat liver. Lipids. (1982) 17:598–604. 10.1007/BF02535365 7144448

